# Comparative evaluation of the effect of simvastatin and modified triple antibiotic paste on microhardness and fracture resistance of root dentin—an *in vitro* study

**DOI:** 10.3389/fdmed.2026.1946257

**Published:** 2026-10-02

**Authors:** Pradeep Kumar, Megha Sreekumar, S. Vidhyadhara Shetty, Nikhil Harikrishnan, Natasha Shetty, Jayaprakash K

**Affiliations:** 1 Department of Consrvative Dentistry and Endodontics, Yenepoya Dental College, Yenepoya Deemed to be University, Mangalore, India; 2 Department of Dental Materials, Yenepoya Dental College, Yenepoya Deemed to be University, Mangalore, India

**Keywords:** dentin microhardness, fracture resistance, modified triple antibiotic paste (mTAP), regenerative endodontics, simvastatin

## Abstract

**Background:**

An ideal intracanal medicament should provide effective antimicrobial action while preserving radicular dentin. This *in vitro* study compared the effects of simvastatin and modified triple antibiotic paste (mTAP) on root dentin microhardness and fracture resistance.

**Materials and methods:**

Fifty-four single-rooted human premolars were randomly assigned to three groups (*n* = 18): simvastatin, mTAP, and control. Following root canal preparation, medicaments were placed for 1 week. Dentin microhardness was measured using a Vickers hardness tester, and fracture resistance was assessed with a universal testing machine. Statistical significance was set at *p* < 0.05.

**Results:**

Both simvastatin and mTAP significantly reduced dentin microhardness compared with baseline (*p* < 0.001). The reduction was greatest with simvastatin (29.84 ± 3.92%), followed by mTAP (17.11 ± 7.04%) and control (8.48 ± 1.76%). Fracture resistance also differed significantly among groups (*p* = 0.0007), with the control group showing the highest values, followed by mTAP and simvastatin.

**Conclusion:**

Both medicaments were associated with changes in mechanical properties of root dentin. Simvastatin showed the greatest reduction in microhardness and the lowest fracture resistance among the tested groups. These findings should be interpreted within the limitations of the *in vitro* experimental model.

## Introduction

The primary objective of endodontic therapy is to eliminate microbial infection from the root canal system, prevent reinfection, promote periapical healing, and preserve the natural tooth. However, complete canal disinfection remains challenging because of the complex root canal anatomy, including lateral canals, isthmus, apical ramifications, and dentinal tubules, which harbor microorganisms beyond the reach of instruments and irrigants. Consequently, intracanal medicaments play a crucial role in reducing the microbial load between treatment visits (1, 2).

An ideal intracanal medicament should possess broad-spectrum antimicrobial activity while preserving the physical properties of root dentin. In addition to disinfection, these medicaments contribute to regenerative endodontic procedures, apexification, and the management of persistent infections. Commonly used medicaments include calcium hydroxide, triple antibiotic paste (TAP), double antibiotic paste (DAP), and Ledermix (3). However, prolonged exposure to certain medicaments has been reported to adversely affect the chemical and mechanical properties of dentin, increasing the risk of structural failure (1).

Simvastatin has recently gained attention in endodontics because of its pleiotropic biological effects beyond lipid lowering. As a semisynthetic analog of lovastatin, it inhibits HMG-CoA reductase and promotes odontogenic differentiation, mineralized tissue formation, antimicrobial activity, biofilm disruption, and reduction of extracellular polymeric substance production (4–6). Schroff et al. also reported that simvastatin demonstrated antibacterial efficacy comparable to double antibiotic paste in reducing intracanal bacterial counts (7).

Modified triple antibiotic paste (mTAP) was developed to overcome the limitations of conventional TAP, particularly tooth discoloration and cytotoxicity associated with minocycline. By replacing minocycline with alternative antibiotics, mTAP exhibits potent activity against resistant endodontic pathogens, especially *Enterococcus faecalis*, while demonstrating reduced cytotoxicity toward stem cells of the apical papilla, making it a promising medicament for regenerative endodontics (8, 9).

Preserving dentin integrity is equally important for the long-term success of endodontic treatment. Dentin microhardness reflects its mineral content and structural integrity; reductions in microhardness indicate demineralization that may compromise the adhesion of restorative materials and increase susceptibility to fracture (10). Likewise, fracture resistance is a critical determinant of the longevity of endodontically treated teeth, as vertical root fractures often result in tooth loss (11). Therefore, an ideal intracanal medicament should effectively eliminate microorganisms while maintaining the mechanical properties of root dentin.

Although the antimicrobial efficacy of simvastatin and mTAP has been investigated, limited evidence is available regarding their effects on dentin microhardness and fracture resistance. Therefore, this *in vitro* study was designed to evaluate and compare the effects of simvastatin and modified triple antibiotic paste on the microhardness and fracture resistance of root dentin, with the aim of identifying an intracanal medicament that provides effective antimicrobial action while preserving dentin structural integrity.

## Materials and methods

This *in vitro* experimental study was approved by the Yenepoya Ethics Committee-2, Yenepoya (Deemed to be University), Mangaluru (Protocol No. YEC2/2026/080). The study conformed to relevant institutional and national research guidelines.

The *in vitro* study aimed to assess and compare the influence of simvastatin and mTAP on the microhardness and fracture resistance of root dentin. The sample size for this study was calculated by using G*Power software program for one-way analysis of variance (ANOVA) with a significance level of 5%, statistical power of 80%, pooled standard deviation of 2.20, and effect size of 0.4427. Therefore, the minimum number of samples used in this study would be 54, with 18 teeth in each group (3, 16).

Fifty-four anonymized, de-identified single-rooted maxillary and mandibular premolars extracted for orthodontic or periodontal reasons were obtained from the Department of Oral and Maxillofacial Surgery. As the study used only previously extracted teeth with no identifiable personal data or human participant involvement, the Institutional Ethics Committee waived the requirement for ethical approval. Single-rooted maxillary and mandibular premolars with a mature apex (complete root formation), free of caries, cracks or fractures, internal or external resorption, calcification, severe curvature, or previous endodontic treatment were selected for the study (1, 3).

Following extraction, residual soft tissue and debris were meticulously removed using an ultrasonic scaler. All specimens were subsequently processed in accordance with Occupational Safety and Health Administration (OSHA) standards and preserved in 0.1% thymol solution until further experimental procedures (13).

### Root canal preparation

The crowns of all specimens were sectioned perpendicular to the long axis, 0.5 mm apical to the cementoenamel junction (CEJ), using a low-speed diamond disc under continuous irrigation. Standardized endodontic access cavities were subsequently prepared with an Endo Access bur. Canal patency was verified with a size #10 K-file, and the working length was established by advancing the file until its tip was visible at the apical foramen, followed by subtracting 0.5 mm from this measurement.

Root canal instrumentation was performed using the ProTaper Gold rotary system up to an F3 apical size. During preparation, canals were irrigated with 2 mL of 3% sodium hypochlorite after each file. Upon completion, a final rinse was carried out with 5 mL of sterile saline delivered through a 27-gauge irrigation needle (16, 18).

### Preparation of dentin specimens

Following root canal preparation, the 54 specimens were numbered sequentially and allocated to the three groups (*n* = 18 each) using a computer-generated random number sequence (e.g., Microsoft Excel RAND function/random number table), ensuring equal allocation to the Simvastatin, mTAP, and Control groups.

The decoronated segments were then sectioned horizontally in order to obtain 2 radicular dentin discs. Fracture resistance assessment was performed on the 5-mm section cervical to the CEJ and microhardness evaluation was made on the middle third 3-mm root section (3).

The specimens were allocated into three groups of 18 specimens each according to the intracanal medicament (ICM) used:.

Group I: Treated with Simvastatin as intracanal medicament
Group II: Treated with modified triple antibiotic paste (mTAP) as intracanal medicament
Group III: Control group (no medicament)

### Preparation and application of intracanal medicaments

### Preparation of simvastatin

The pre-measured quantity of methyl cellulose was dissolved in an adequate amount of biocompatible solvent (1 mL of distilled water) at a temperature of 50–60 °C by continuous stirring until a clear and homogenous solution was obtained. Simvastatin was then added to the methylcellulose-based polymer solution and stirred until completely dissolved to obtain a homogeneous *in situ* gel containing 1.2% (w/v) simvastatin (26).

### Preparation of modified triple antibiotic paste

Following the peeling off of the enteric coating, the metronidazole (400 mg), clindamycin (300 mg), and ciprofloxacin (500 mg) tablets were crushed separately in a sterile mortar and pestle. Equal weights of each powdered antibiotic (as obtained from the crushed tablets) were then combined in the ratio 1:1:1 and mixed in 1 mL of sterile distilled water, and stirred for 2 h on a hot plate magnetic stirrer for complete dissolution. Thereafter, 4% methylcellulose was used as a carrier and stabilizing medium, and the mixture was stirred further for 2 h at 40–50 °C to obtain a homogeneous modified triple antibiotic paste (mTAP), achieving a final concentration of 1 mg/mL (8, 9).

Following preparation, the intracanal medicaments were introduced into the prepared root canals using a Lentulo spiral. 0.1 mL of the respective intracanal medicament was applied to each specimen, ensuring that the entire surface of the root dentin was covered. The same method of medicament placement was followed for all specimens in the respective experimental groups. Once the medicaments were placed, all specimens were incubated at 37 °C and 100% relative humidity for one week to maintain specimen hydration and simulate the intraoral environment. The schematic representation of the experimental workflow is depicted in Figure 1. The one-week exposure period was selected to approximate the interval commonly used for intracanal medicament placement between treatment appointments, while providing sufficient contact time for interaction between the medicaments and radicular dentin and allowing measurable changes in its mechanical properties (15, 25).

**Figure 1:**
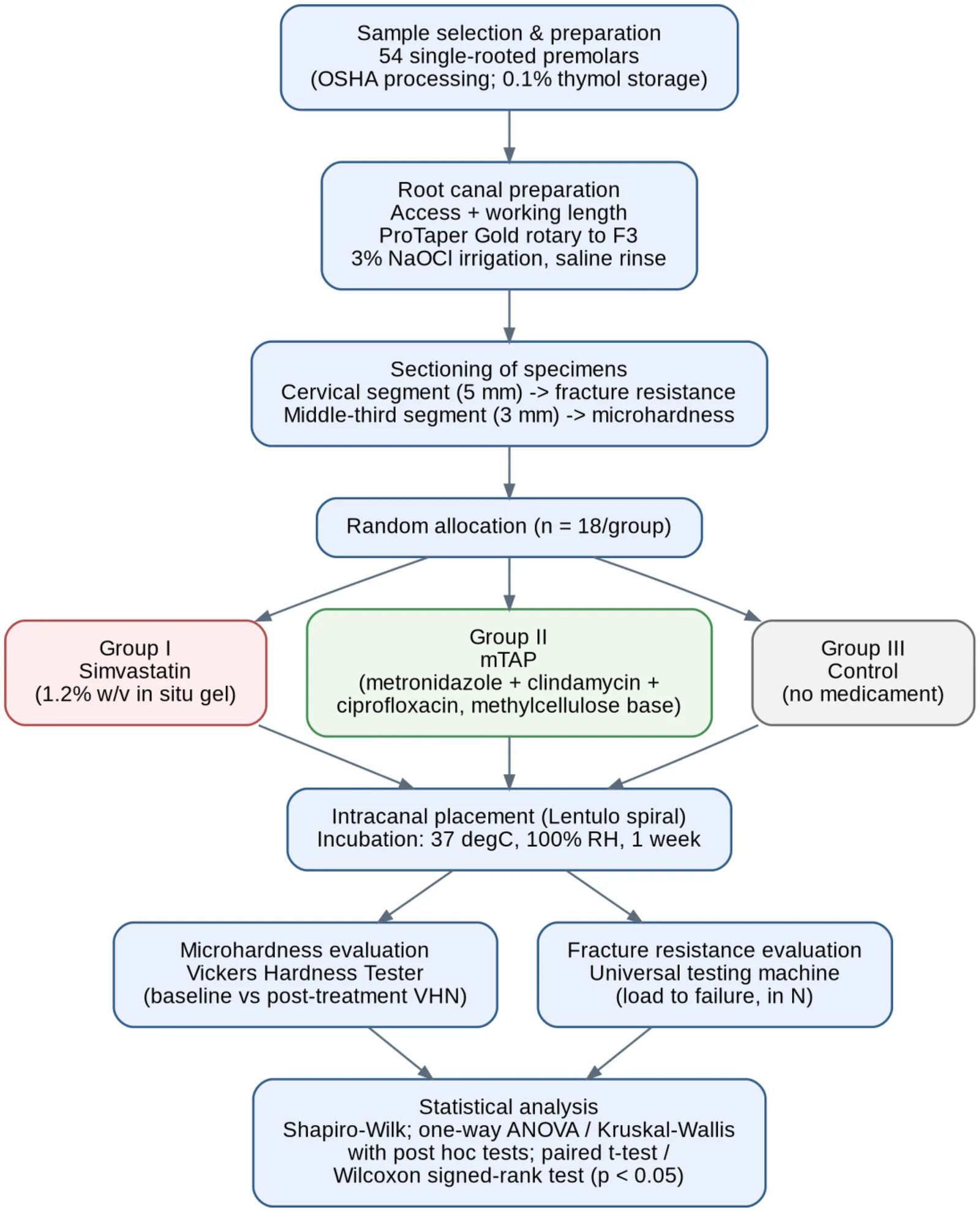
Schematic illustration of the experimental workflow.

### Microhardness evaluation

Following the incubation period, the specimens were rinsed with distilled water to remove any residual medicament and were then dried in air prior to testing. The specimen segments were smoothened using a mounted fine-grained grinding stone and further treated with silicon carbide abrasive paper, ranging from 600 to 2,000 grit, to obtain a polished surface prior to Vickers microhardness testing (1).

The microhardness of root dentin was measured after one week of incubation using the Vickers Microhardness Tester (Tecsol, India) (Figure 2B). Three indentations were made using an indenter stylus with a 50 g load applied for 15 s, held perpendicular to the polished surface of the root dentin, and the mean of the three values was recorded for each specimen. The indentations were positioned at a standard distance of 0.5 mm from the canal wall in the mid-root dentin, ensuring consistency across all samples. The mean VHN of each sample was determined thereafter. The impact of the intracanal medicaments on the microhardness properties of root dentin was evaluated through this process (1).

**Figure 2:**
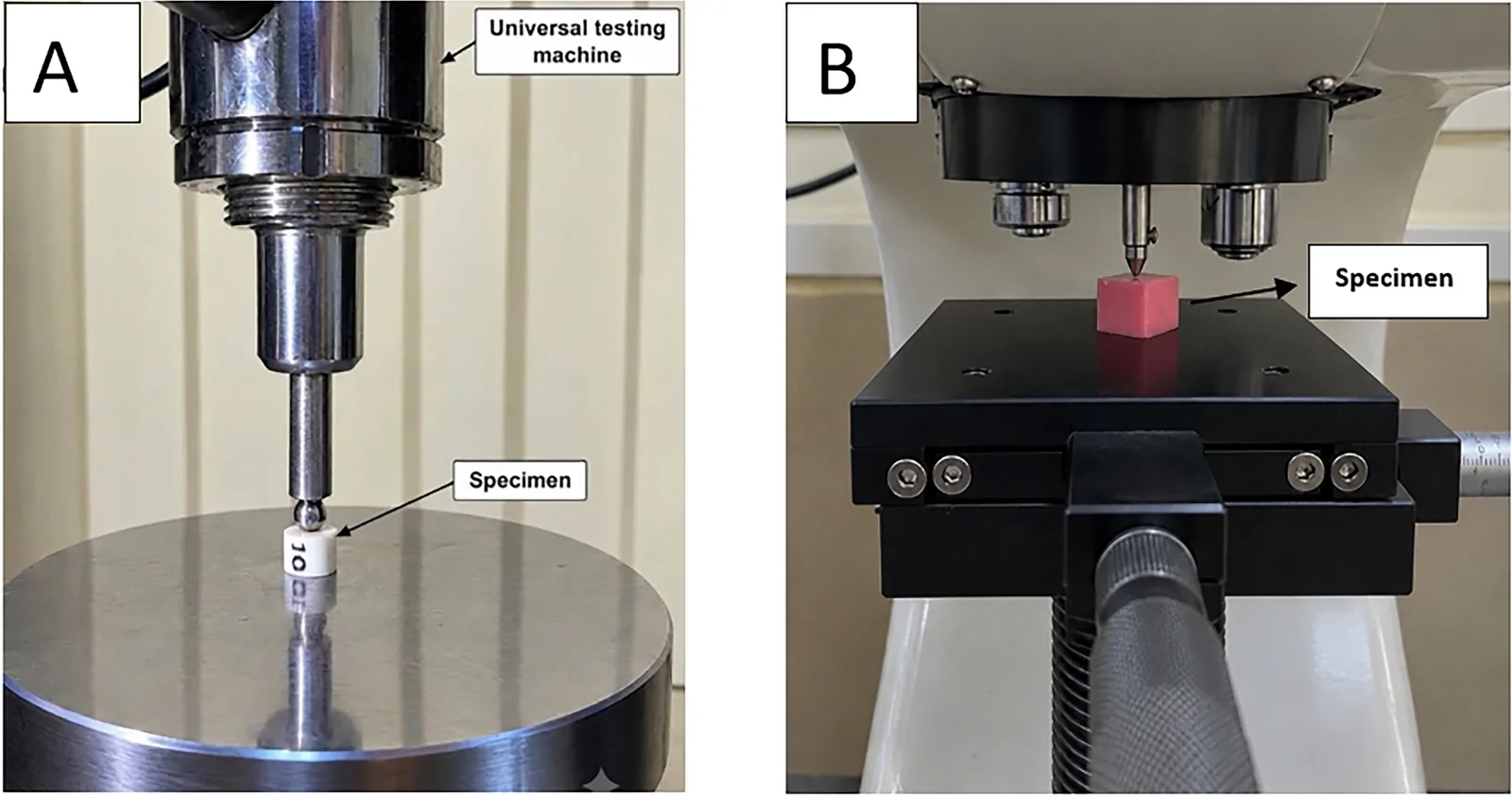
**(A)** universal testing machine. **(B)** Vickers microhardness tester.

### Fracture resistance evaluation

Fracture resistance was tested using a universal testing machine (Tecsol, India) (Figure 2A). Each specimen was positioned with the coronal side facing upward, vertically on the lower platform of the universal testing machine. A cylindrical tip with a radius of 1.9 mm was fixed to the upper plate and positioned until it contacted the specimen. A loading force was applied at a crosshead speed of 0.5 mm/min until the root specimen fractured, and the load at fracture was recorded in Newtons (1). Fracture/failure was defined as the point at which a sharp and instantaneous drop greater than 25% of the applied load was observed (27).

#### Blinding

The operator responsible for medicament placement was not blinded in this *in vitro* study. However, the outcome assessor performing the microhardness and fracture resistance measurements was blinded to group allocation, using coded/anonymized specimen labels that did not reveal group assignment.

## Statistical analysis

Data were compiled in Microsoft Excel and statistically analyzed using IBM SPSS Statistics version 27.0 (IBM Corp., Armonk, NY, USA) and Jamovi software. The normality of data distribution was evaluated using the Shapiro–Wilk test. Quantitative data were presented as mean ± standard deviation (SD) for normally distributed variables and as median with interquartile range (IQR) for non-normally distributed variables. Comparisons among multiple groups were performed using one-way analysis of variance (ANOVA) followed by appropriate *post hoc* tests for parametric data, whereas the Kruskal–Wallis test was applied for non-parametric data. Intra group comparisons between baseline and post-treatment measurements were conducted using paired t-tests or Wilcoxon signed-rank tests, as appropriate. A *p*-value of less than 0.05 was considered statistically significant. To further account for baseline differences among groups, an analysis of covariance (ANCOVA) was performed on post-treatment microhardness values with baseline microhardness as a covariate. Normality of distribution was additionally assessed separately for percentage reduction in microhardness and for fracture resistance in each group using the Shapiro–Wilk test; where this assumption was not met, findings were corroborated using the Kruskal–Wallis test. Effect sizes (Cohen's d) and 95% confidence intervals were calculated for pairwise group comparisons of fracture resistance and for baseline-adjusted microhardness comparisons.

## Results

### Microhardness evaluation

All groups demonstrated a statistically significant reduction in dentin microhardness after a 1-week incubation period (*p* < 0.001). The greatest reduction was observed in the Simvastatin group, while the Control group exhibited the least reduction. (Table 1).

**Table 1:** Comparison of dentin microhardness before and after 1 week.

Group (*n* = 18)	Before (Mean ± SD)	After 1 week (Mean ± SD)	Mean difference	Paired t-test *p*-value
mTAP	46.48 ± 6.15	38.27 ± 3.23	8.21 ± 4.18	<0.001*
Simvastatin	45.16 ± 2.67	31.64 ± 1.15	13.52 ± 1.62	<0.001*
Control	59.57 ± 1.25	54.61 ± 1.84	4.96 ± 0.71	<0.001*

* Statistically significant. mTAP, modified triple antibiotic paste; SD, standard deviation.

Percentage reduction was calculated after 1 week of incubation using the formula:

Percentage reduction = [(Pre-treatment microhardness−Post-treatment microhardness)/Pre-treatment microhardness] × 100.

A highly significant difference in percentage reduction of dentin microhardness was observed among the three groups (*p* < 0.001). The Control group exhibited the least percentage reduction in microhardness (8.48 ± 1.76%), indicating superior preservation of dentin hardness. The Simvastatin formulation was associated with significantly greater reductions in microhardness (29.84 ± 3.92%) than mTAP (17.11 ± 7.04%) (Table 2). *Post hoc* pairwise comparisons (Tukey's honestly significant difference test) showed that the percentage reduction in microhardness differed significantly between every pair of groups: Simvastatin versus Control (*p* < 0.001), Simvastatin versus MTAP (*p* < 0.001), and MTAP versus Control (*p* < 0.001). This confirms that simvastatin, mTAP, and the control group each represented a distinct level of effect on dentin microhardness, rather than the overall ANOVA result being driven by a difference between only one pair of groups. To account for the baseline differences among groups, an ANCOVA was additionally performed on post-treatment microhardness values with baseline microhardness as a covariate. The covariate was significantly associated with post-treatment microhardness [F(1,50) = 30.91, *p* < 0.001], and a significant effect of group persisted after adjustment [F(2,50) = 139.90, *p* < 0.001]. Baseline-adjusted mean microhardness values were 51.08 (Control), 39.25 (MTAP), and 33.54 (Simvastatin) VHN. Pairwise comparisons of adjusted means showed that MTAP differed from Control by −11.83 (95% CI: −13.94 to −9.73, *p* < 0.001), Simvastatin differed from Control by −17.54 (95% CI: −19.73 to −15.36, *p* < 0.001), and Simvastatin differed from MTAP by −5.71 (95% CI: −6.90 to −4.53, *p* < 0.001). Given non-normal distribution of percentage-reduction values on Shapiro–Wilk testing (*p* < 0.001 for all groups), findings were corroborated using the Kruskal–Wallis test (H = 40.84, *p* < 0.001), which confirmed the ANOVA result.

**Table 2:** Comparison of percentage reduction in dentin microhardness Among groups.

Group (*n* = 18)	Percentage Reduction (%) (Mean ± SD)
mTAP	17.11 ± 7.04
Simvastatin	29.84 ± 3.92
Control	8.48 ± 1.76

mTAP, modified triple antibiotic paste; SD, standard deviation.

### Fracture resistance evaluation

A statistically significant overall difference in fracture resistance was observed among the three groups (*p* = 0.0007). The mean fracture resistance was 1445.39 ± 108.44 N for the control group, 1382.87 ± 121.35 N for the mTAP group, and 1290.45 ± 118.26 N for the simvastatin group. The control group demonstrated the highest mean fracture resistance, followed by mTAP and simvastatin (Table 3). Pairwise comparisons (Tukey's HSD) with effect sizes (Cohen's d) and 95% confidence intervals were: Simvastatin vs. Control, mean difference 154.94 N, d = 1.37, 95% CI 78.08 to 231.80, *p* = 0.0006 (significant); mTAP vs. Simvastatin, mean difference 92.42 N, d = 0.77, 95% CI 11.26 to 173.58, *p* = 0.053 (not significant); mTAP vs. Control, mean difference 62.52 N, d = 0.54, 95% CI −15.43 to 140.47, *p* = 0.249 (not significant). Full *post hoc* results are presented in Tables 4 and 5.

**Table 3:** Fracture resistance comparison Among groups.

Group (*n* = 18)	Mean ± SD (*N*)
mTAP	1382.87 ± 121.35
Simvastatin	1290.45 ± 118.26
Control	1445.39 ± 108.44

N, newton; mTAP, modified triple antibiotic paste; SD, standard deviation.

**Table 4:** *Post hoc* pairwise comparisons (tukey's HSD)—percentage reduction in dentin microhardness.

Comparison	Mean difference (%)	Cohen's d	95% CI	*p*-value	Significance
Simvastatin vs. Control	21.36	7.03	19.30 to 23.42	<0.001	Significant
Simvastatin vs. mTAP	12.73	2.23	8.87 to 16.59	<0.001	Significant
mTAP vs. Control	8.63	1.68	5.15 to 12.11	<0.001	Significant

mTAP, modified triple antibiotic paste; HSD, honestly significant difference; CI, confidence interval.

**Table 5:** *Post hoc* pairwise comparisons (tukey's HSD)—fracture resistance.

Comparison	Mean difference (*N*)	Cohen's d	95% CI	*p*-value	Significance
Simvastatin vs. Control	154.94	1.37	78.08 to 231.80	0.0006	Significant
mTAP vs. Simvastatin	92.42	0.77	11.26 to 173.58	0.053	Not significant
mTAP vs. Control	62.52	0.54	−15.43 to 140.47	0.249	Not significant

N, newton; mTAP, modified triple antibiotic paste; HSD, honestly significant difference; CI, confidence interval.

## Discussion

The selection of an intracanal medicament requires consideration of both antimicrobial efficacy and preservation of radicular dentin, particularly when prolonged medication is used in regenerative endodontic procedures (12, 13). In the present study, both simvastatin and modified triple antibiotic paste (mTAP) adversely affected dentin mechanical properties after one week, with simvastatin producing a greater reduction in microhardness and fracture resistance. These findings suggest that the therapeutic potential of intracanal medicaments should be balanced against their effects on dentin integrity.

The observed changes are consistent with Ghosh et al., who reported that intracanal medicaments can reduce the mechanical strength of radicular dentin, with the extent of deterioration influenced by medicament type and exposure duration (12). Although different mechanical parameters were assessed, the findings collectively indicate that chemical exposure during intracanal medication may compromise dentin stability. This is clinically relevant when medicaments are retained for extended periods.

Dentin microhardness is influenced by the mineralization and integrity of the hydroxyapatite–collagen matrix and is therefore considered an indirect indicator of changes in dentin mineral content (14). The reduction observed with mTAP agrees with previous reports linking antibiotic-containing medicaments with dentin demineralization and alterations in mineral composition (15, 16). Thakur et al. reported a substantial reduction in microhardness following mTAP application, while Amonkar et al. attributed such changes to the acidic nature of antibiotic formulations and their ability to dissolve dentinal mineral (3, 16). Similar reductions following prolonged intracanal medication have also been reported by Halkai et al. (1). The comparatively smaller effect of mTAP in the present study may be related to the absence of minocycline, which has greater calcium-chelating capacity and may promote more pronounced demineralization (17). Acid-mediated dissolution of calcium and phosphate ions and disruption of the mineral–organic interface have been proposed as possible mechanisms (17, 18).

Interestingly, simvastatin produced a greater reduction in microhardness than mTAP, despite its established antimicrobial and regenerative properties. Simvastatin has been shown to promote odontoblastic differentiation, BMP-2 expression, alkaline phosphatase activity, and mineralization of dental pulp stem cells (19). However, these biological effects do not necessarily indicate preservation of existing mineralized dentin. Diffusion into dentinal tubules (20), possible alterations in calcium dynamics and collagen–mineral interactions (5), and effects related to the methylcellulose-based vehicle may contribute to the observed changes. Because a vehicle-only control was not included, the individual contribution of simvastatin and methylcellulose cannot be determined. Elgamal et al. reported intermediate microhardness values with simvastatin compared with mTAP nanofibers, with differences potentially attributable to variations in formulation, vehicle, exposure duration, and methodology (18). Thus, the mechanical effects of these medicaments appear to be formulation- and protocol-dependent.

The fracture-resistance findings broadly paralleled the microhardness results, with the simvastatin group demonstrating the greatest reduction. Fracture resistance is influenced by dentin structure, hydration, collagen cross-linking, and mineralization (21), and disruption of the hydroxyapatite–collagen framework may impair load distribution and increase susceptibility to fracture (22). Previous studies have similarly associated antibiotic-containing medicaments and prolonged exposure to acidic formulations with reductions in dentin fracture resistance (3, 11). However, because no correlation analysis was performed in the present study, a direct relationship between microhardness and fracture resistance cannot be established.

The present findings provide a biomechanical perspective to the existing evidence supporting simvastatin's antimicrobial and regenerative potential. Simvastatin has demonstrated inhibition of endodontic biofilm formation (4), antimicrobial activity comparable to double antibiotic paste (7), and promotion of odontogenesis and mineralized tissue formation (5, 6). Nevertheless, its effects on acellular dentin may differ from its biological effects in living tissues (23), while extracted teeth lack the reparative mechanisms present *in vivo* (24). Therefore, the observed reduction in dentin mechanical properties should be interpreted as a potential consideration rather than evidence against the clinical use of simvastatin.

Several limitations warrant consideration. The *in vitro* design, single concentration and formulation, one-week exposure period, modest sample size, and absence of blinding limit generalizability. The lack of a vehicle-only control prevents isolation of the effect of methylcellulose, while the absence of direct chemical or ultrastructural analyses limits interpretation of the underlying mechanisms. Furthermore, antimicrobial, cytotoxic, regenerative, and mechanical effects were not assessed simultaneously. Future studies should therefore investigate different formulations, concentrations, exposure periods, and aging conditions using techniques such as SEM, EDS, FTIR, Raman spectroscopy and microCT, followed by *in vivo* and clinical evaluation.

## Conclusion

Within the limitations of this *in vitro* study, one-week exposure to simvastatin and modified triple antibiotic paste was associated with changes in the measured mechanical properties of root dentin. Simvastatin demonstrated the greatest reduction in microhardness and the lowest mean fracture resistance among the tested groups, whereas mTAP showed intermediate values. Although an overall difference in fracture resistance was observed among the groups, the present findings do not establish a direct causal relationship between medicament exposure and tooth fracture susceptibility. Further studies incorporating vehicle-only controls, different concentrations and exposure periods, structural and chemical analyses, and clinically relevant loading conditions are required to determine the significance of these findings.

## Data Availability

The original contributions presented in the study are included in the article/Supplementary Material, further inquiries can be directed to the corresponding author.
